# Thermal-Resistance Effect of Graphene at High Temperatures in Nanoelectromechanical Temperature Sensors

**DOI:** 10.3390/mi13122078

**Published:** 2022-11-26

**Authors:** Shuai Lei, Ningning Su, Mengwei Li

**Affiliations:** 1 Academy for Advanced Interdisciplinary Research, North University of China, Taiyuan 030051, China; 2 State Key Laboratory of Dynamic Measurement Technology, North University of China, Taiyuan 030051, China; 3 School of Semiconductors and Physics, North University of China, Taiyuan 030051, China

**Keywords:** graphene, temperature sensor, temperature coefficient of resistance, substrate

## Abstract

Graphene membranes act as temperature sensors in nanoelectromechanical devices due to their excellent thermal and high-temperature resistance properties. Experimentally, reports on the sensing performance of graphene mainly focus on the temperature interval under 400 K. To explore the sensing performance of graphene temperature sensors at higher temperature intervals, micro-fabricated single-layer graphene on a SiN_X_ substrate is presented as temperature sensors by semiconductor technology and its electrical properties were measured. The results show that the temperature coefficient of the resistance value is 2.07 × 10^−3^ in the temperature range of 300–450 K and 2.39 × 10^−3^ in the temperature range of 450–575 K. From room temperature to high temperature, the “metal” characteristics are presented, and the higher TCR obtained at higher temperature interval is described and analyzed by combining Boltzmann transport equation and thermal expansion theory. These investigations provide further insight into the temperature characteristics of graphene.

## 1. Introduction

Accurately monitoring temperature with small size has become a critical role not only in life-threatening ailments but also in modern industries with the increasing maturity of micromachining and chip integration technology. Compared with conventional temperature sensors, micro-temperature sensors have attracted research attention because of their miniaturization [1], intelligence [2], and high integration [3,4]. Graphene exhibits excellent properties such as high electron mobility [5], low noise [6], and impressive thermal properties [7,8]. Graphene exhibits the highest thermal conductivity of about 5300 W·m^−1^·K^−1^ among the currently known materials [9,10]. Graphene relies on electrons and phonons for heat transfer [11], but the main heat transfer medium is phonons [12]. For this reason, graphene is an ideal material for heat-sensitive elements.

The temperature characteristics of graphene-based sensitive elements such as reduced graphene oxide (rGO) [13,14] and graphene film below 400 K have been reported [15]. However, with the development of technology and the prosperity of industrialization, there is an increasing demand for miniaturized thermal resistance microsensors for high-temperature testing [16,17,18]. For example, in the process of petroleum exploration and development, the measurement of formation temperature, which determines the properties of oil and gas and the way of gas field development, needs to reach above 500 K [18]. In the pharmaceutical process, the monitoring temperature of drug production and sterilization can reach above 530 K [17]. So it is highly necessary to improve the temperature measurement interval of the thermal resistance temperature sensor with high sensitivity. It has been reported that graphene, as an excellent high-temperature resistant material, can maintain stable performance at 973K [19] and exhibits “metallic” characteristics in the temperature range around room temperature because of phonon scattering [20]. However, as a zero-bandgap semi-metallic material, the charge carriers may be excited to participate in the conduction at high temperature, thus, the transition from “gold” to “semiconductor” will occur. To the best of our knowledge, no experimental reports have focused on the temperature characteristics of graphene at high temperatures. Therefore, it is of profound physical significance to explore the scientific question of whether graphene temperature sensors exhibit monotonic properties at high temperatures. In addition, Benyamin Davaji et al. confirmed that the temperature sensor with SiN substrate exhibits the highest temperature coefficient by comparing the sensitivity of micro-fabricated single-layer graphenes (SLGS) on a SiO_2_/Si substrate, SiN membrane and a suspended architecture [21]. Therefore, a graphene temperature sensor with SiN_X_/Si substrate to explore the temperature characteristics of graphene in a wider temperature range was fabricated in this study.

In this article, graphene prepared by chemical vapor deposition (CVD) was developed to a thermal resistance nanoelectromechanical graphene temperature sensor by using standard semiconductor processes such as transferring and sputtering. The temperature characteristics of graphene were tested in the temperature range of 300–575 K, and its temperature characteristics were explained by combining Boltzmann theory and thermal expansion theory. Experimental support for the development of temperature sensors with graphene as a sensitive element in a higher range will be provided in this study. Simultaneously, the previously proposed model was improved from the perspective of thermal expansion of the substrate, and the concept that the tensile strain caused by thermal expansion of the substrate intensifies the “metallic” property of graphene was explained. In the temperature interval studied here, the increase of the positive temperature coefficient (PTC) could be described as the superposition of the dominant scattering mechanism of graphene itself and the tensile strain of the external substrate.

## 2. Materials and Methods

**Figure 1:**
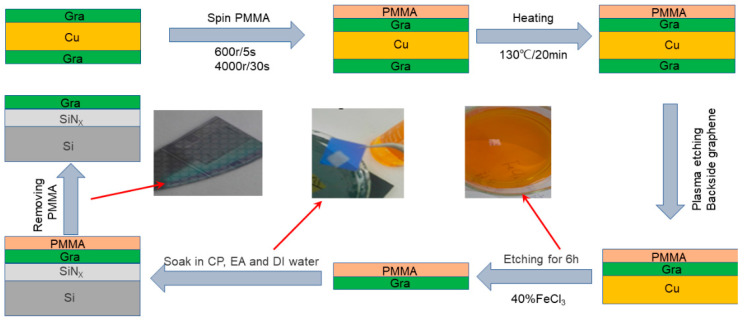
Schematic diagram of graphene transferring process.

Graphene was grown on copper substrate by the chemical vapor deposition (CVD) method [22]. A wet transfer method using polymethyl methacrylate (PMMA) as a support layer was used to transfer graphene onto a SiN_X_/Si substrate (made by plasma-enhanced chemical vapor deposition (PECVD)) pre-deposited with metal electrodes [23,24]. The process of graphene being transferred to the specified substrate is presented in Figure 1. After being cropped into 1.5 × 1.5 cm samples, a spin coater was used to apply graphene to copper foil at speeds of 600 rpm/5 s and 4000 rpm/30 s and performed with polymethyl methacrylate (PMMA). PMMA/Graphene/Cu/Graphene samples were placed on a hot plate at 130 °C for 20 min for hot plate hardening to allow PMMA to bind more tightly to the graphene after curing. Next, the samples were treated with O_2_ plasma to etch the back graphene of the sample, and 40% FeCl_3_ was used to etch the Cu substrate supporting the graphene for about 6 h. After that, the PMMA/graphene sample was transferred to the target substrate and heated on a hot plate at 85 °C for 30 min. The PMMA layer was dissolved in acetone (CP) solution at 50 °C for 10 min, and then the target was washed with alcohol (EA) solution and deionized (DI) water. Finally, The graphene was patterned by using lithography and O_2_ plasma etching. The parameters of O_2_ plasma etching are as follows: power, 60 W; gas flow, 30 sccm; and etching time 3 min.

**Figure 2:**
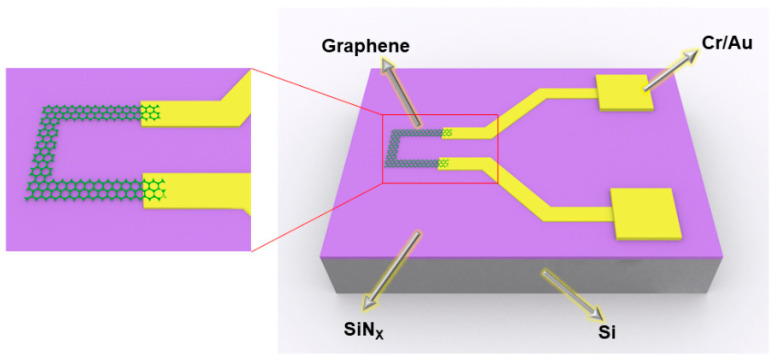
The structure diagram of the graphene temperature sensor.

The structure of the graphene temperature sensor is shown in Figure 2. 15 nm Cr was deposited as the adhesion layer by UV lithography and magnetron sputtering on the Si/SiNx substrate, and then 25 nm Au was deposited as the bottom electrode, which was connected with the transferred monolayer graphene. The sputtering rate of Cr and Au was 1.26 nm/s and 2.35 nm/s, respectively. The figure on the left is a local magnification of the sensitive unit graphene. The graphene is green, and the connecting wire and electrode are yellow.

**Figure 3:**
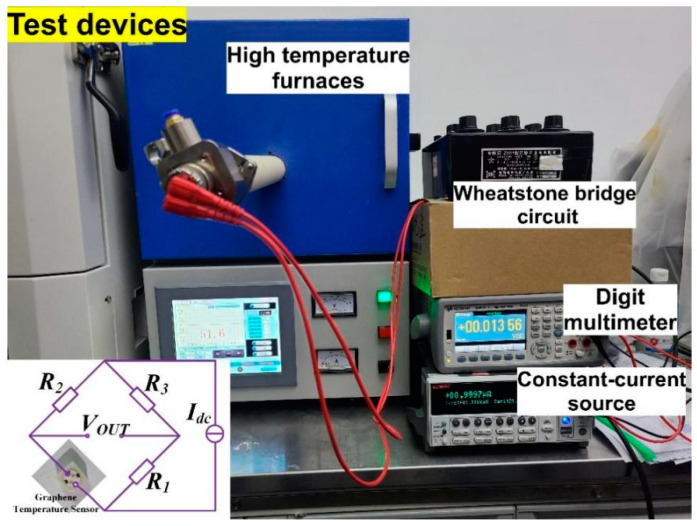
Field test diagram.

After the graphene temperature sensor device was fabricated, the temperature characteristic of the graphene temperature sensor was tested by using a high-temperature sealed tube and a high-temperature furnace combined with a Wheatstone bridge. Figure 3 shows the test diagram of the temperature sensor, where the sensor was placed in a high-temperature sealed tube in a high-temperature furnace. The test temperature was increased from 300 K to 575 K in 25 K increments and was maintained at each temperature for 10 min to stabilize the temperature inside the furnace. In addition, to mitigate the humidity and gas sensitivity effects of the graphene film, the high-temperature furnace was evacuated and repeatedly cleaned with inert nitrogen gas prior to the measurement to ensure its oxygen-free environment.

To accurately measure the change in resistance during the heating process, the device resistance was measured using a Wheatstone bridge (see illustration). The bridge was equilibrated down at room temperature, where R_0_ was equal to the initial resistance of the sensor at room temperature with a humidity of 45% to 50%. The voltage at both ends of the bridge changed linearly with the change of resistance. The output voltage signal was amplified by an operational amplifier, sampled by a low-pass filter, and finally converted to the corresponding resistance value.

## 3. Results and Discussion

**Figure 4:**
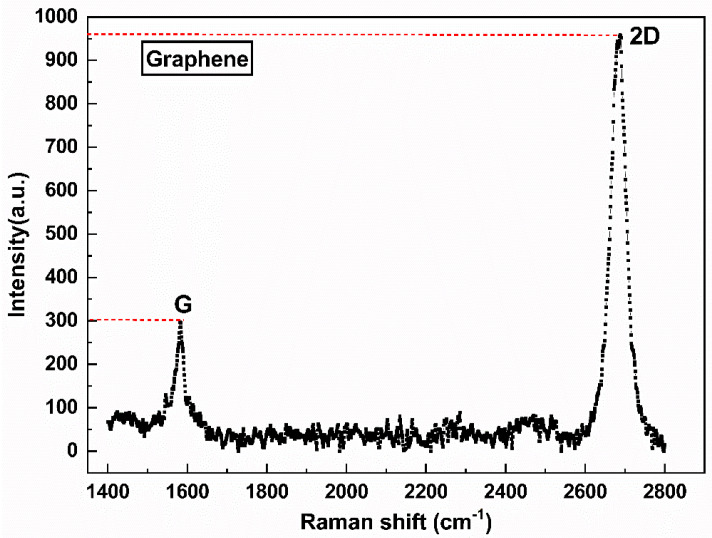
Raman spectra of graphene.

To characterize the number of layers and defects of graphene after transferring, samples were subjected to Raman scattering (HR-800 Horiba Scientific, Inc., Paris, France) [25]. As shown in Figure 4, the G and 2-D peaks are located at 1582 cm^−1^ and 2700 cm^−1^, respectively, and the intensity ratio of the 2-D peak to the G peak (I_2D_/I_G_) is 3.21, which indicates that the transferred graphene is a monolayer film [26]. No D peak representing defects appeared at 1350 cm^−1^, which indicated that the graphene film was of high quality [27], and the defects introduced into graphene during the transferring process could be ignored.

**Figure 5:**
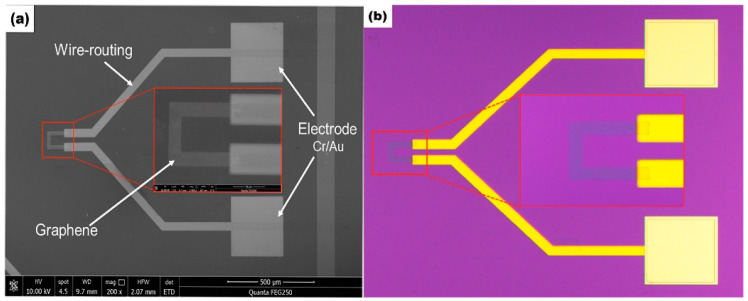
(**a**) SEM image of the chip. (**b**) Optical microscope image of the chip.

The electrical and thermal conductivity of graphene would be weakened because of the presence of cracks, folds, and organic residues on the graphene surface [28,29,30,31], thus affecting the performance of sensor components. Therefore, to understand the morphology and quality of the graphene surface in the experiment, it was characterized by scanning electron microscopy (Quanta 250, FEI, Inc., Hillsboro, OR, USA) and light microscopy (OM, Axio Scope, Carl Zeiss AG, Oberkochen, Germany). Figure 5a shows the SEM image of the graphene temperature sensor chip, and it can be seen that the transferred graphene surface is smooth and wrinkle-free. Figure 5b shows the chip image of the graphene temperature sensor under the light microscope. From the enlarged area, it can be seen that there is less organic matter residue, no cracks, and a high density of electrode deposition. These results indicated that the transferring quality of graphene films in this experiment was relatively high, and the effect of cracks, folds, and residues on subsequent measurement results could be ignored.

**Figure 6:**
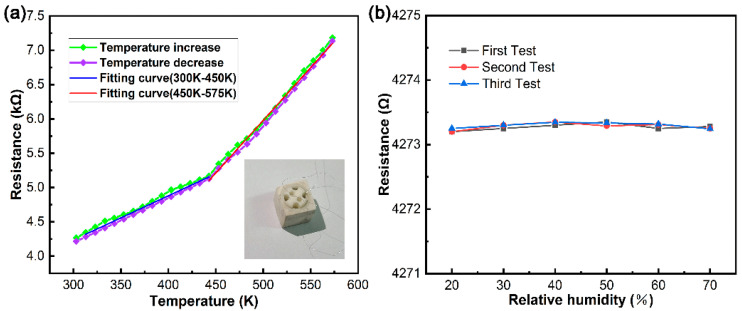
(**a**) The resistance of the temperature sensor as a function of temperature. (**b**) Humidity characteristics of the device.

Figure 6a shows the curve of resistance with temperature in the range of 300 to 575 K. The experimental result shows a positive temperature coefficient and the “metal” characteristics because the resistance increases with the increase in temperature, and the resistance decreases with the decrease in temperature. This could be attributed to the dominant phonon scattering in this temperature range, and the enhancement of phonon scattering with the increase in temperature is caused by the decrease in mobility [32,33]. The resistance does not increase linearly with the increase in temperature, which could be attributed to the existence of multiple physical mechanisms in the graphene transport process, such as phonons, screening, Fermi surface effects, and carrier activation, etc., which are all temperature-dependent [34]. Hwang et al. used Boltzmann theory to calculate the relationship between the resistance and temperature of graphene under phonon scattering, which was expressed as ρ ~ T [35], and the resistivity and temperature revealed a linear relationship. Any mechanisms had an impact on the temperature performance of graphene, not only screening that resulted in the “metal” behavior but both the Fermi surface effects and the carrier activation that produced the semiconductor behavior. In the presence of multiple scattering mechanisms, the temperature behavior of graphene depended on the dominant scattering mechanism. Therefore, the relationship between resistance and temperature was not simply summarized as the superposition relationship of multiple scattering mechanisms, ρ_tol_(T) ≠ ρ_PH_(T) +ρ_other_(T). ρ_tol_(T) represented the temperature characteristic that graphene exhibits as resistance varies with temperature. ρ_PH_(T) represented the temperature characteristic exhibited by phonon scattering, and ρ_other_(T) represented all the scattering mechanisms except phonon scattering. This statement explains the non-linear experimental result in the experiment. The humidity test of the device was carried out, and the results are shown in Figure 6b. The results show that the resistance of the encapsulated device is almost not affected by the relative humidity, which indicates that the device can be used normally under different relative humidity environments, and also shows the high reliability of the device.

The temperature coefficient of resistance (TCR) is one of the performance indicators of a temperature sensor. To characterize the performance of the sensor, the TCR can be calculated according to Equation (1). As could be visible in Figure 6, the resistance increased with the increase in temperature, but the slope exhibited an inflection point around 450 K. Therefore, we calculated the TCR in the temperature interval of 300–450 K and 450–475 K, respectively:

(1) $$TCR=\frac{1}{R_{0}}\cdot \frac{R-R_{0}}{T-T_{0}}$$

where *R*_0_ represents the resistance value at initial temperature *T*_0_ and *R* is the resistance value at temperature T.

The TCR at 300–450 K temperature can be calculated as follows: TCR_1_ = (5.52425 − 4.2155)/(4.2155·(450 − 300)) = 2.07 × 10^−3^. The TCR at 450–575 K temperature range can be calculated as follows: TCR_2_ = (7.13572 − 5.52425)/(5.52425·(575 − 450)) = 2.39 × 10^−3^.

After calculation, the TCR of the high-temperature interval is greater than that of the lower-temperature interval. One possible reason is that during the heating process, graphene is subjected to tensile strain from the substrate because SiN_X_ expands with increasing temperature. As a result, the resistance of graphene gradually increases during tensile strain, which indirectly aggravates the “metal” characteristics of graphene, presenting a higher temperature coefficient.

Table 1 shows temperature ranges and TCR for different materials as sensitive junctions are compared. The results show that the graphene temperature sensor has a high-temperature range and high TCR value.

## 4. Conclusions

A graphene temperature sensor with a temperature range from 300 K to 573 K was fabricated by using standard semiconductor integration technology. Scanning electron microscopy, optical microscopy, Raman analysis revealed that graphene is a single layer of defect-free structure with a clean surface. An electrical performance test using a wheatstone bridge revealed that temperature sensor resistance increased with the increase of temperature, and the reason is that the phonon scattering dominates the presence of the characteristics of “metal” in the temperature range. Simultaneously, the TCR of the high-temperature interval was higher, a possible reason for enhancing this characteristic was explained. In brief, the temperature characteristic of graphene in the range of 300 to 574 K was investigated, and a wide range, good linearity, and high sensitivity graphene temperature sensor has been fabricated, which meets the needs of industrial production and other fields for accurate temperature control at high temperatures.

## Figures and Tables

**Table 1:** Comparison of different materials’ temperature characteristics.

Materials	Temperature (K)	TCR (K^−1^)
Silicon rubber-CB, CNTs [36]	293–353	0.00572
PVDF-MWCNTs, PEN [37]	295–373	0.081
RGO [14]	303–343	−1.7 × 10^−3^
Graphene ink [15]	213–333	−1.55 × 10^−3^ to −1.02 × 10^−3^
Laser-induced-graphene [38]	294–354	0.573 × 10^−3^
Metallurgical graphene [39]	6–360	−1.5 × 10^−4^
Graphene (CVD) [This work]	300–573	2.39 × 10^−3^

## Data Availability

Not applicable.
